# News and Notes: *Emerging Infectious Diseases* Is Moving to Online Only

**DOI:** 10.3201/eid2512.NN2512

**Published:** 2019-12

Starting with the January 2020 issue, *Emerging Infectious Diseases* (EID) will join the growing ranks of journals published online only. We made the decision to stop publishing on paper with the recognition that our readers increasingly access the journal only online, and not through paper copies. In addition, we think the move offers at least three advantages to the journal and its readers. First, we can use budget dollars saved for other important journal functions, such as editing and production. EID is now recruiting a new assistant editor, who will help speed up the review of submitted manuscripts.

Second, we can “go green.” Printing and mailing paper issues of the journal carry environmental costs. In recent years, we have come to believe that these costs are not outweighed by whatever advantages remain to printed pages.

Third, we can place even more emphasis on online-only materials included as supplements or appendices to articles published in the journal. These materials now represent a substantial portion of all the pages that we publish. We think that, in the future, they will become an even more important part of the journal.

Readers should rest assured that EID articles will continue to be available online as they have before, along with supplemental materials and appendices. Entire issues of the journal will continue to be available in the PDF format. Readers who have enjoyed browsing a full printed issue of EID can continue to so by using any Web-connected desktop or laptop computer, tablet, or smartphone.

We invite all readers to subscribe to our monthly table of contents alerts on the journal’s Web site at https://wwwnc.cdc.gov/eid/subscriptions.

D. Peter Drotman, MD, MPH

Editor in Chief

